# The Reliability of the Mediterranean Diet Quality Index (KIDMED) Questionnaire

**DOI:** 10.3390/nu9040419

**Published:** 2017-04-23

**Authors:** Lovro Štefan, Rebeka Prosoli, Dora Juranko, Marko Čule, Ivan Milinović, Dario Novak, Goran Sporiš

**Affiliations:** 1 Faculty of Kinesiology, University of Zagreb, 10 000 Zagreb, Croatia; rebeka.prosoli@kif.hr; 2 Boutique Fitness Studio “Vježbaonica”, Center for Recreation and Fitness, 10 000 Zagreb, Croatia; dora.juranko@kif.hr; 3 Faculty of Economics and Business, University of Zagreb, 10 000 Zagreb, Croatia; mcule@efzg.hr (M.Č.); imilinovic@efzg.hr (I.M.); 4 Faculty of Kinesiology, University of Zagreb, 10 000 Zagreb, Croatia; dario.novak@kif.hr (D.N.); goran.sporis@kif.hr (G.S.)

**Keywords:** Mediterranean diet, questionnaire, nutrition, young adults, reliability

## Abstract

The purpose of the present study was to determine the test–retest reliability of the Mediterranean Diet Quality Index (KIDMED) questionnaire in college students. Two hundred and seventy-six college students (127 men, 46%; 149 women, 54%; mean age 19.70 ± 1.32 years; mean height 1.75 ± 0.09 m; mean weight 69.28 ± 13.84 kg; mean body-mas index 22.41 ± 3.19 kg/m^2^) participated in the study. To investigate the reliability of the KIDMED questionnaire, the participants were asked to complete the questionnaire on two occasions two weeks apart, stratified by gender. Kappa statistics showed moderate to excellent agreement (ranging from 0.504 to 0.849) in the total sample and moderate to excellent agreement in both men (ranging from 0.467 to 0.803) and women (ranging from 0.435 to 0.927). Results in the total KIDMED score showed a moderate correlation between two occasions inthe total sample (κ = 0.597, *p* < 0.001) and in women (κ = 0.586, *p* < 0.001) and a good correlation in men (κ = 0.611, *p* < 0.001). Our study shows that the KIDMED questionnaire is a reliable instrument for assessing adherence to the Mediterranean diet in college students. Future studies should focus on investigating the reliability of the questionnaire in other countries and in different age groups for generating comparable data.

## 1. Introduction

The Mediterranean diet is often characterized by a high consumption of fruits, vegetables, legumes, nuts, cereals, and olive oil, a moderateto high intake of fish, dairy products, and alcohol (whichcomes primarly from wine), and a low intake of saturated lipids, sweets, and red and processed meat [1]. It represents the dietary pattern consumed by the populations situated near the Mediterranean sea, and several studies have shown that such a diet has beneficial effects against cardiovascular [2,3,4], metabolic [5,6,7], and mental diseases [8,9].

In children and youth, the adherence to the Mediterranean diet has often been investigated in the Mediterranean region [10,11,12,13,14,15,16,17]. For example, the prevalence of children and adolescents having low adherence to the Mediterranean diet was 4.2% in Spain [14], 14.9% and 27% for children and adolescents in Greece [15,16], andbetween 23.0% and 33.0% in Italy [17,18]. Based on these results, the geographical region seems to play animportant role for the higher adherence, whereas Mediterranean countries do not always show the highest adherence to the Mediterranean diet. Most recently, one study showed that the prevalence of children and adolescents from non-mediterranean European countries having average adherence to the Mediterranean diet was roughly 48% and around 13% for high adherence [19]. In the United States, one recent study showed that, to the Mediterranean diet, almost 81.4% of adolescents had poor adherence, while 17.8% showed average adherence and only 0.75% of them good adherence [20]. In general, the prevalence of average to high adherence to the Mediterranen diet is between 40% and 50% in children and adolescents, while adherence decreases with age.

Young adulthood is characterized by great changes, such as self-development and identity, and by increased levels of autonomy in decision-making [21] and defined as a period between 18–25 years of age [21]. People in that age tend to have a nutrient-poor diet, often accompanied by a high consumption of fast food and sugar-sweetened beverages [22]. Moreover, food choice is often accompanied by socioeconomic status. Socioeconomic status has been shown to have positive association on adherence to the Mediterranean diet. Specifically, youth who have parents on a higher socioeconomic level tend to have higher adherence to the Mediterraean diet [19]. Since diet qualitycan affect future risk of a number of chronic diseases at later ages, it is necessary to establish the reliability properties of the appropriate diet assessing tool, especially designed for young adults [14]. Most recently, one study has shown that the KIDMED questionnaire is one of the most widely used scoring systems to assess adherence to the Mediterranean diet [23]. However, to the best of author’s knowledge, based on an extensive literature review, there has been no study investigating the reliability properties of the KIDMED questionnaire. Thus, the main purpose of the present study was to determine the test–retest reliability of the KIDMED questionnaire in college students. 

## 2. Materials and Methods

### 2.1. Participants and Study Protocol

For the purpose of this study, participants werea convinience sample of the first and second year from the Faculty of Economics and Business from the city of Zagreb, Croatia. Of 350 participants initially selected for the study, 300 of them were randomly selected to complete the KIDMED questionnaire to determine its reliability. Randomization of students was done with replacement by drawing student codes on slips of paper from a box, with each student having equal probability of selection. All the participants included in the analysis were healthy without any medical conditions.Basic descriptive statistics of the study participants are presented in Table 1. The appropriate sample size was at minimum 220 participants, taking into account a moderate effect size (*w* = 0.3), an α level of 0.05 and a statistical power of 0.95 (1 − β). Twenty-four participants were excluded from further analysis because of data inconsistency or absence from the class. At the end, 276 participants (127 men, 46%; 149 women, 54%) were included in the analysis. The questionnaire was given on two occasions, at the baseline and after a two-week period. A two-week period is a sufficient amount of time to eliminate the influence of the first test responses on the results of the retest [24]. Before the study began, all participants and their parents/guardians had given informed consent to participate in the study. Additionally, all participants were toldthat the research was voluntary and participants could withdraw at any time. KIDMED questionnaire was translated to Croatian by two experienced and independent experts who were familiar with the questionnaire. Than, potential disagreements and differences in the process of translation were removed and a third independent expert translated the Croatian-type questionnaire back into English language to determine the accuracy and quality of translation [25]. The testing procedure was conducted from February to March 2017. Each participant was given specific instruction on how to completethe questionnaire. The questionnaire was completedin small groups of 10–15 people. Each participant got a specific number (code) on the questionnaire and the whole procedure was anonymous. After completing the questionnaire, each questionnaire was put inside the box. The same procedure was done on the second occasion. All procedures performed in this study were anonymous and in accordance with Declaration of Helsinki, also approved by the Institutional Review Board of the Faculty of Kinesiology, University of Zagreb, Croatia. 

### 2.2. The KIDMED Questionnaire

The KIDMED questionnairewas used to evaluate the adherence to a Mediterranean diet in adolescents. It consists of 16 items, where there are 4 questions denoting a negative connotation to the Mediterranean diet (consumption of fast food, baked goods, sweets, and skipping breakfast) and 12 questions denoting a positive connonation (consumption of oil, fish, fruits, vegetables, cereals, nuts, pulses, pasta or rice, dairy products, and yoghurt). Questions denoting negative connotation are scored with −1, while positive connotation questions are scored with +1. According to the KIDMED index, a score of 0–3 reflects poor adherence to the Mediterranean diet, a score of 4–7 describes average adherence, and a score of 8–12 good adherence [14]. The same questionnaire has been previously used in the college student population [26,27]. Additional variables were body height, weight, and calculated body-mass index by using a formula: weight (kg)/height (m^2^). Additionally, we asked about educational level of both parents and categorized it into fivecategories: (1) primary school, (2) secondary school, (3) faculty (bachelor degree), (4) faculty (master’s degree), and (5) faculty (PhD).

### 2.3. Data Anaysis

The data obtained by both men and women were analyzed by using Statistical Packages for Social Sciences software ver. 23 (SPSS, Chicago, IL, USA). Numerical variables are presented as means (±standard deviations) and categorical variables are presented as frequencies (*N*) and percentages (%). The Kolmogorov–Smirnov test was used to determine the data normality distribution. Since all numerical-type variables had normally distributed data and there was no homogeneity of variance violation, differences between men and women were analyzed by using a Student’s *t*-test for independent samples. Differences in categorical variables were analyzed by using a chi-square test. To assess the reliability of the KIDMED questionnaire, we used frequencies and percentages for each question within the questionnaire and correlations between two occasions were analyzed using Kappa statistics. Kappa values range between −1 (perfect disagreement) and +1 (perfect agreement). Kappa values ≤ 0.20, 0.21 to 0.40, 0.41 to 0.60, 0.61 to 0.80, and 0.81 to 1.00 represent poor, fair, moderate, good, and excellent agreement. To determine the differences between the two time occasions in categorical variables, we used the McNemar test. First, we calculated the McNemar test and Kappa statistics values for each of 16 questions within the questionnaire. Next, we calculated KIDMED score and performedthe same calculations in the total sample and stratified by gender. Significance was set up at α ≤ 0.05 and was two-sided.

## 3. Results

Basic descriptive statistics of the study participants are presented in Table 1. Men were significanly taller and heavier than women, which led to higher values of body-mass index (*p* < 0.001). Interestingly, most of the participants reported secondary school as the highest education level of their parents, but no differences occurred between gender (*p* = 0.140 and *p* = 0.220). Additionally, additional analysis showed (data not presented) that higher adherence to the Mediterranean diet in college students was significantly associated with both the mother’s (*r* = 0.14, *p* = 0.020) and the father’s (*r* = 0.17, *p* = 0.005) educational level.

Basic descriptive statistics of the total sample in the KIDMED questionnaire are presented in Table 2. The highest prevalence was in consumption of fruit or fruit juice daily (81.5%), use of olive oil at home (roughly 74.0%), and consumption of dairy products for breakfast (75.7%) at the baseline. Two weeks later, participants reported similar values in almost every question, but the highest prevalence remained in consumption of fruit or fruit juice daily (82.2%), the use of olive oil at home (74.3%), and consumption of dairy products for breakfast (77.9%).There were no significant changes in question responses between the first and second occasion, only inthe question referred to consumption of pasta or rice almost daily (≥5 days/week), where 31.2% of the participant reported having pasta or rice almost daily, yet 37.3% of them reported having pasta or rice almost daily on the second occasion (*p* = 0.016). Kappa statistics showed moderate to excellent agreement in each question (ranging between 0.504 and 0.849), while good agreement was obtained in the KIDMED index score(κ = 0.597, *p* < 0.001).

Basic descriptive statistics of men in the KIDMED questionnaire are presented in Table 3. Similar to the results in the total sample, the highest prevalence was reported in consumption of fruit or fruit juice daily (78.0%), the use of olive oil at home (71.7%), and consuming dairy products for breakfast (69.3%). After two weeks, somewhat higher percentages were reported in the consumption of fruit or fruit juice daily (80.3%) and the use of olive oil at home (73.2%). As in the total sample, significant changes in frequencies occurred in consumption of pasta or rice almost daily (≥5 days/week), where 32.3% of men reported having pasta or rice almost daily, yet 42.5% of them reported consuming pasta or rice almost daily (≥5 days/week) on the second occasion (*p* = 0.011). However, no significant differences occurred between the first and second occasions in other questions (*p* > 0.05) and almost 30.0% of men reported having fast-food (hamburger) > 1 day/week in arestaurant. Additionally, 30.0% of them reported having no breakfast and 53.5% of them reported consuming baked goods for breakfast. Kappa statistics showed moderate to good agreement between the two occasions (ranging between 0.467 and 0.803). By looking at the KIDMED index score, Kappa statistics showed good agreement between the two occasions (κ = 0.611, *p* < 0.001).

Results in Table 4 show basic descriptive statistics of women in the KIDMED questionnaire. Similar to the results from the total sample and men, the highest prevalence was reported in consumption of fruit or fruit juice daily (84.6%), the use of olive oil at home (75.2%) and consuming dairy products for breakfast (85.2%). Interestingly, women had a statistically higher prevalence of fruit, olive oil, and dairy product consumption than men. On the second occasion, 83.9% of them reported consuming fruit or fruit juice daily, 75.2% reported using olive oil at home and 85.2% of them reported eating dairy products for breakfast. Similar to men, 26.2% of women reported going into restaurant for fastfood (hamburger) > 1/week, while a somewhat higher percentage (35.6%) of them reported having no breakfast. Kappa statistics showed moderate to good agreement between the two occasions (ranging between 0.435 to 0.927). By looking at the KIDMED index score, Kappa statistics showed moderate agreement between the two occasions (κ = 0.586, *p* < 0.001).

## 4. Discussion

The main purpose of the present study was to determine the test–retest reliability of the KIDMED questionnaire in young adults. To the best of our knowledge, this is the first study examining the reliability properties of the KIDMED questionnairein college students.

We found moderate to good agreement between the two occasions ofeach question the and general score of theKIDMED questionnaire. The only significant difference occurred in the question referring to the consumptionof pasta or rice almost daily (≥5 days/week). The transition from secondary school to college or university represents a critical period for young adults, where they are facing great challenges making their own food decisions, which may lead to a negative impact on eating behaviors [28]. Previous findings have shown that college students often have poor eating habits, often accompanied by eating fewer fruits and vegetables on a daily basis and higher consumption of high-fat, high-calorie foods [29]. For example, one study showed that only 13.0% of college students reported having a second serving of fruit and vegetables at least two times per day, while 50.0% of them consumed no fruits and vegetables on a daily basis [30]. Additionally, another study reportedthat roughly 66.0% of college students were not eating fruits and vegetables daily [31]. Our results showed that both men and women reported consuming dairy products for breakfast. However, one previous study showed that the intake of dairy products for breakfast reduced by 0.5 servings in both men and women during the transition period between middle adolescence and young adulthood [32]. Additionally, one study conducted on college students from Cyprus showed that approximately 50.0% of them reported consuming pulses more than once a week [33], which is consistent with the results from our study. Next, our results showed that 30.0% of the total sample consumed sweets and candy several times a day, which is similar to previous studies conducted on college students [33,34]. In general, about 30.0% of college students report consuming sweets and candy several times a day, with a somewhat higher percentage in women [33]. Additionally, previous findings have shown that around 25% of college students reported going to afast-food restaurant more than once a week, concluding that fast-food consumption, along with skipping breakfast, could lead to increased weight gain from childhood to adulthood [35].

Our main analysis showed moderate to excellent agreement between two measurements two weeks apart. The only significant difference occurred in the consumption of pasta or rice almost daily (≥5 days/week) in both men and women. This great range by each question could be explained by the fact that college students, due to their residence, have different eating habits and diet-related health [36]. For example, one study concluded that students living off campus tended to choose different types of food opposed tothosestudents living on campus, since thosewho live in campuses often eat in cafeterias [36]. Additionally, another potential factor associated with food choice is money. One study has shown that Mediterranean products cost more than Western products (products richwith fatty acids, high-calorie diet), so groups with lowersocioeconomic statuscannot not afford such diet products and often reach for nutrient-poor diet [37].

Our study has several limitations. We only included students from one faculty. Since there are different professions (e.g., medical doctors) who often deal with a high amount of stress, future studies should take other students from a variety of professions into account, to determine the test–retest reliability of the KIDMED questionnaire. To the best our knowledge and after an extensive literature review, this is the first study examining the test–retest reliability of the KIDMED questionnaire in college students. Thus, the current study is especially important, because it shows the use of the KIDMED questionnaire in college students.

## 5. Conclusions

In conclusion, the main purpose of the present study was to determine the test–retest reliability of the widely used KIDMED questionnaire for assessing the adherence to the Mediterranean diet. To the best of our knowledge, this is the first study investigating the test–retest reliability of the KIDMED questionnaire in college students. Our results show that the questionnaire has adequate test–retest reliability, and it can be used in the population of college students. As mentioned, future studies should take special emphasis on investigating the reliability and validity of the questionnaire in other college students populations for generating comparable data between groups and other countries.

## Figures and Tables

**Table 1:** Basic descriptive statistics of the study participants expressed as the mean (±standard deviation) or number (%).

Study Variables	Total Sample (*N* = 277)	Men (*N* = 129)	Women (*N* = 149)	*p*-Value
*Numerical variables*				
Age (years)	19.70 ± 1.32	19.76 ± 1.42	19.64 ± 1.24	0.456
Body height (m)	1.75 ± 0.09	1.82 ± 0.06	1.69 ± 0.06	<0.001
Body weight (kg)	69.28 ± 13.84	79.98 ± 11.10	60.17 ± 8.33	<0.001
Body-mass index (kg/m^2^)	22.41 ± 3.19	24.01 ± 3.00	21.05 ± 2.69	<0.001
*Categorical variables*				
**Mother’s education**				
Primary school	23 (8.3)	11 (8.7)	12 (8.1)	
Secondary school	138 (50.0)	56 (44.1)	82 (55.0)	
Faculty (Bachelor degree)	51 (18.5)	25 (19.7)	26 (17.4)	
Faculty (Master’s degree)	59 (21.4)	33 (26.0)	26 (17.4)	
Faculty (PhD)	5 (1.8)	2 (1.6)	3 (2.0)	0.140
**Father’s education**				
Primary school	15 (5.5)	6 (4.8)	9 (6.1)	
Secondary school	170 (62.0)	77 (61.1)	93 (62.8)	
Faculty (Bachelor degree)	31 (11.3)	12 (9.5)	19 (12.8)	
Faculty (Master’s degree)	50 (18.2)	25 (19.8)	25 (16.9)	
Faculty (PhD)	10 (3.0)	7 (4.8)	3 (1.4)	0.220

**Table 2:** Mediterranean Diet Quality Index statistics for total sample (*N* = 276).

Study Variables	Total (*N* = 276)				McNemar Test (*p*-Value)	Kappa Statistics (*p*-Value)
	Baseline		After 2 Weeks			
	Yes	No	Yes	No		
	*N* (%)	*N* (%)	*N* (%)	*N* (%)		
Fruit or fruit juice daily	225 (81.5)	51 (18.5)	227 (82.2)	49 (17.8)	0.845	0.683 (<0.001)
Second serving of fruit daily	116 (42.0)	160 (58.0)	117 (42.4)	159 (57.6)	1.000	0.725 (<0.001)
Fresh or cooked vegetables daily	141 (51.1)	135 (48.9)	143 (51.8)	133 (48.2)	0.892	0.608 (<0.001)
Fresh or cooked vegetables > 1/day	46 (16.7)	230 (83.3)	43 (15.6)	233 (84.4)	0.743	0.504 (<0.001)
Regular fish consumption (at least 2–3/week)	48 (17.4)	228 (82.6)	53 (19.2)	223 (80.8)	0.500	0.576 (<0.001)
>1/week fast-food (hamburger) restaurant	76 (27.5)	200 (72.5)	68 (24.6)	208 (75.4)	0.256	0.643 (<0.001)
Pulses > 1/week	131 (47.5)	145 (52.5)	137 (49.9)	139 (50.4)	0.480	0.638 (<0.001)
Pasta or rice almost daily (≥5 days/week)	86 (31.2)	190 (68.8)	103 (37.3)	173 (62.7)	0.016	0.639 (<0.001)
Cereal or cereal product for breakfast	137 (49.6)	139 (50.4)	146 (52.9)	130 (47.1)	0.175	0.746 (<0.001)
Regular nut consumption (at least 2–3/week)	107 (38.8)	169 (61.2)	111 (40.2)	165 (59.8)	0.678	0.606 (<0.001)
Use of olive oil at home	203 (73.6)	73 (26.4)	205 (74.3)	71 (25.7)	0.832	0.793 (<0.001)
No breakfast	95 (34.4)	181 (65.6)	98 (35.5)	178 (64.5)	0.648	0.849 (<0.001)
Dairy product for breakfast	209 (75.7)	67 (24.3)	215 (77.9)	61 (22.1)	0.362	0.695 (<0.001)
Commercially baked goods or pastries for breakfast	149 (54.0)	127 (46.0)	154 (55.8)	122 (44.2)	0.487	0.759 (<0.001)
Two yoghurts and/or 40 g cheese daily	103 (37.3)	173 (62.7)	112 (40.6)	164 (59.4)	0.253	0.627 (<0.001)
Sweets and candy several times a day	92 (33.3)	184 (66.7)	87 (31.5)	189 (68.5)	0.473	0.744 (<0.001)
*KIDMED index score*						
Poor (≤3)	118 (42.8)		114 (41.3)		0.063	0.597 (<0.001)
Average (4–7)	129 (46.7)		121 (43.8)			
Good (≥8)	29 (10.5)		41 (14.9)			

**Table 3:** Mediterranean Diet Quality Index statistics for men (*N* = 127).

Study Variables	Men (*N* = 127)				McNemar Test (*p*-Value)	Kappa Statistics (*p*-Value)
	Baseline		After 2 Weeks			
	Yes	No	Yes	No		
	*N* (%)	*N* (%)	*N* (%)	*N* (%)		
Fruit or fruit juice daily	99 (78.0)	28 (22.0)	102 (80.3)	25 (19.7)	0.581	0.690 (<0.001)
Second serving of fruit daily	62 (48.8)	65 (51.2)	57 (44.9)	70 (55.1)	0.359	0.700 (<0.001)
Fresh or cooked vegetables daily	56 (44.1)	71 (55.9)	63 (49.6)	64 (50.4)	0.230	0.606 (<0.001)
Fresh or cooked vegetables > 1/day	21 (16.5)	106 (83.5)	22 (17.3)	105 (82.7)	1.000	0.580 (<0.001)
Regular fish consumption (at least 2–3/week)	18 (14.2)	109 (85.8)	22 (17.3)	105 (82.7)	0.481	0.467 (<0.001)
>1/week fast-food (hamburger) restaurant	37 (29.1)	90 (70.9)	33 (26.0)	94 (74.0)	0.481	0.645 (<0.001)
Pulses > 1/week	72 (56.7)	55 (43.3)	70 (55.1)	57 (44.9)	0.832	0.649 (<0.001)
Pasta or rice almost daily (≥5 days/week)	41 (32.3)	86 (67.7)	54 (42.5)	73 (57.5)	0.011	0.618 (<0.001)
Cereal or cereal product for breakfast	54 (42.5)	73 (57.5)	55 (43.3)	72 (56.7)	1.000	0.727 (<0.001)
Regular nut consumption (at least 2–3/week)	44 (34.6)	83 (65.4)	47 (37.0)	80 (63.0)	0.678	0.606 (<0.001)
Use of olive oil at home	91 (71.7)	36 (28.3)	93 (73.2)	34 (26.8)	0.754	0.803 (<0.001)
No breakfast	42 (33.1)	85 (66.9)	44 (34.6)	83 (65.4)	0.791	0.754 (<0.001)
Dairy product for breakfast	88 (69.3)	39 (30.7)	88 (69.3)	39 (30.7)	1.000	0.704 (<0.001)
Commercially baked goods or pastries for breakfast	68 (53.5)	59 (46.5)	73 (57.5)	54 (42.5)	0.332	0.729 (<0.001)
Two yoghurts and/or 40 g cheese daily	60 (47.2)	67 (52.8)	60 (47.2)	67 (52.8)	1.000	0.621 (<0.001)
Sweets and candy several times a day	35 (27.6)	92 (72.4)	33 (26.0)	94 (74.0)	0.791	0.719 (<0.001)
*KIDMED index score*						
Poor (≤3)	55 (43.3)		51 (40.2)		0.281	0.611 (<0.001)
Average (4–7)	58 (45.7)		56 (44.1)			
Good (≥8)	14 (11.0)		20 (15.7)			

**Table 4:** Mediterranean Diet Quality Index statistics for women (*N* = 149).

Study Variables	Women (*N* = 149)				McNemar Test (*p*-Value)	Kappa Statistics (*p*-Value)
	Baseline		After 2 Weeks			
	Yes	No	Yes	No		
	*N* (%)	*N* (%)	*N* (%)	*N* (%)		
Fruit or fruit juice daily	126 (84.6)	23 (15.4)	125 (83.9)	24 (16.1)	1.000	0.672 (<0.001)
Second serving of fruit daily	54 (36.2)	95 (63.8)	60 (40.3)	89 (59.7)	0.238	0.745 (<0.001)
Fresh or cooked vegetables daily	85 (57.0)	64 (43.0)	80 (53.7)	69 (46.3)	0.458	0.607 (<0.001)
Fresh or cooked vegetables > 1/day	25 (16.8)	124 (83.2)	21 (14.1)	128 (85.9)	0.523	0.435 (<0.001)
Regular fish consumption (at least 2–3/week)	30 (20.1)	119 (79.9)	31 (20.8)	118 (79.2)	1.000	0.650 (<0.001)
>1/week fast-food (hamburger) restaurant	39 (26.2)	110 (73.8)	35 (23.5)	114 (76.5)	0.503	0.641 (<0.001)
Pulses > 1/week	59 (39.6)	90 (60.4)	67 (45.0)	82 (55.0)	0.185	0.616 (<0.001)
Pasta or rice almost daily (≥5 days/week)	45 (30.2)	104 (69.8)	49 (32.9)	100 (67.1)	0.523	0.658 (<0.001)
Cereal or cereal product for breakfast	83 (55.7)	66 (44.3)	91 (61.1)	58 (38.9)	0.096	0.752 (<0.001)
Regular nut consumption (at least 2–3/week)	63 (42.3)	86 (57.7)	64 (43.0)	85 (57.0)	1.000	0.602 (<0.001)
Use of olive oil at home	112 (75.2)	37 (24.8)	112 (75.2)	37 (24.8)	1.000	0.784 (<0.001)
No breakfast	53 (35.6)	96 (64.4)	54 (36.2)	95 (63.8)	1.000	0.927 (<0.001)
Dairy product for breakfast	121 (81.2)	28 (18.8)	127 (85.2)	22 (14.8)	0.180	0.665 (<0.001)
Commercially baked goods or pastries for breakfast	81 (54.4)	68 (45.6)	81 (54.4)	68 (45.6)	1.000	0.784 (<0.001)
Two yoghurts and/or 40 g cheese daily	43 (28.9)	106 (71.1)	52 (34.9)	97 (65.1)	0.108	0.615 (<0.001)
Sweets and candy several times a day	57 (38.3)	92 (61.7)	54 (36.2)	95 (63.8)	0.629	0.756 (<0.001)
*KIDMED index score*						
Poor (≤3)	63 (42.3)		63 (42.3)		0.282	0.586 (<0.001)
Average (4–7)	71 (47.7)		65 (43.6)			
Good (≥8)	15 (10.1)		15 (10.1)			
